# Minimal Clinically Important Difference and Patient Acceptable Symptom State for Patient-Reported Outcomes Following Common Arthroscopic Sports Surgeries of the Knee: A Systematic Review

**DOI:** 10.1177/23259671251403143

**Published:** 2026-01-13

**Authors:** Dusan Kovacevic, Gurjovan Sahi, Darius L. Lameire, Aazad Abbas, Daniel B. Whelan, John Theodoropoulos, Tim Dwyer, Jaskarndip Chahal

**Affiliations:** † Temerty Faculty of Medicine, University of Toronto, Toronto, ON, Canada; ‡ Division of Orthopaedic Surgery, Department of Surgery, University of Toronto, Toronto, ON, Canada; § University of Toronto Orthopaedic Sports Medicine, University of Toronto, Toronto, ON, Canada; ‖ Division of Orthopaedic Surgery, Women's College Hospital, Toronto, ON, Canada; ¶ Division of Orthopaedic Surgery, St. Michael's Hospital, Toronto, ON, Canada; # Division of Orthopaedic Surgery, Mount Sinai Hospital, Toronto, ON, Canada; Investigation performed at the Division of Orthopaedic Surgery, Department of Surgery, University of Toronto, Toronto, ON, Canada

**Keywords:** knee, arthroscopy, PROMs, MCID, PASS

## Abstract

**Background:**

Patient-reported outcome measures (PROMs) are widely used to capture patients’ perspectives when evaluating outcomes after arthroscopic knee surgery. To aid in the interpretation of PROM scores at the individual level, the minimal clinically important difference (MCID) and patient acceptable symptom state (PASS) are used. These metrics provide patient-centered thresholds for what constitutes a meaningful improvement and satisfactory state postoperatively.

**Purpose:**

To summarize available literature on MCID and PASS values for all PROMs across common primary arthroscopic knee sports surgeries.

**Study Design:**

Systematic review; Level of evidence, 4.

**Methods:**

MEDLINE, Embase, and Cochrane databases were queried from inception through January 13, 2025, to identify studies that calculated MCID or PASS values of PROMs after primary knee arthroscopic surgeries of the anterior cruciate ligament (ACL), meniscus, or cartilage. Study characteristics, MCID and PASS thresholds, and threshold calculation methods were extracted. MCID and PASS thresholds were aggregated by PROM, surgical treatment, and calculation method and then summarized using a range.

**Results:**

In total, 59 studies met the inclusion criteria; 52 studies calculated MCID thresholds, with 15 studies using anchor-based methods and 39 studies using distribution-based methods. A total of 21 studies calculated PASS thresholds; 35 studies calculated thresholds for ACL procedures, 15 studies for meniscus procedures, and 8 studies for cartilage procedures. ACL reconstruction was the most reported procedure (n = 32 studies). Thresholds were calculated for 18 different PROMs, with the International Knee Documentation Committee Subjective Knee Evaluation Form (IKDC) being the most frequently reported instrument (n = 34 studies). The range of MCID thresholds for the IKDC after ACL reconstruction was 7.1 to 16.2 using anchor-based methods and 7.6 to 10.5 using distribution-based methods, while PASS thresholds ranged from 66.7 to 80.5.

**Conclusion:**

The heterogeneity observed in reported MCID and PASS values suggests that these metrics should be viewed as context-specific. While most studies in this review used distribution-based calculations to derive MCID values, anchor-based calculations should be prioritized in future studies as they better reflect the patient's perception of improvement. Overall, this study will allow investigators to use appropriate clinically relevant thresholds for designing randomized controlled trials, comparing the proportion of patients achieving a meaningful improvement and satisfactory state across different treatment arms, and establishing patient expectations for recovery.

Arthroscopic knee surgery is among the most common orthopaedic procedures as it is used to treat many sports injuries, including anterior cruciate ligament (ACL) tears, meniscal injuries, and cartilage damage.^29,41^ Given the high volume of these procedures and patients’ expectations to return to preinjury levels of function,^25,95^ it is important to evaluate whether patients experience noticeable improvements and have acceptable symptoms after surgery.

Patient-reported outcome measures (PROMs) are widely used to capture patients’ perspectives when evaluating outcomes after arthroscopic knee surgery. The integration of PROMs helps to capture individual experiences of pain, satisfaction, and overall quality of life, among other health-relevant concepts.^57^ PROMs have most commonly been expressed as continuous data at the group level (eg, mean, standard deviation), which can be difficult to interpret and translate to the responses of individual patients.^82,89^ To aid in the interpretation of PROM scores at the individual level, the concepts of the minimal clinically important difference (MCID) and patient acceptable symptom state (PASS) have been developed.

The MCID is defined as the smallest change in an outcome that patients perceive as clinically meaningful.^36^ As such, this metric can be used to better understand whether a certain change in score is clinically relevant at the individual level, not just at the statistical level. The MCID reflects the concept of improvement and indicates if a patient is “feeling better,” which requires a pre- and posttreatment assessment of a PROM.

Two common methods to calculate MCID thresholds are anchor-based and distribution-based approaches, although there are many subcategories of calculation methods within these approaches.^1,14,15^ Anchor-based approaches determine the clinical importance of a change by analyzing the smallest change in a PROM that correlates with a meaningful change to the patient, defined through an independent assessment of improvement (ie, in relation to an anchor question).^1^ Distribution-based approaches rely on some measurement of variability in PROM scores, such as standard deviation or effect size, to determine the MCID.^1^ Common distribution-based MCID calculations include half of the standard deviation of a PROM and the standard error of measurement.^14^

Both anchor-based and distribution-based approaches have their own merits and limitations for determining the MCID. Anchor-based approaches allow patients to directly quantify importance; however, they may be limited by the choice of anchor, are vulnerable to recall bias, and fail to account for the precision of the PROM.^1,8,55,62^ Distribution-based approaches, despite their simplicity and common utilization in the literature, are considered less informative because they rely on the statistical properties of the distribution of outcome scores rather than the patient's perception of improvement.^1,8,55,62^ Anchor-based approaches are recommended as the primary tool in calculating the MCID, while distribution-based methods should be considered supportive or secondary measures.^16,62,74^

Although the MCID helps interpret whether a change score is clinically meaningful for a patient, it does not describe whether the patient is satisfied at a given moment, which can be captured using the PASS. The PASS is defined as the score at which patients feel their current state is overall acceptable given their expectations and activity goals.^88^ In contrast to the MCID, the PASS reflects the concept of well-being and “feeling good.” In other words, the PASS is a snapshot into a patient's current state and represents an absolute value of an outcome, rather than a change in score, that serves as a threshold to discriminate between feeling well and feeling unwell.^11^ The cross-sectional approach to assessing PASS achievement means that only posttreatment scores are required, unlike the longitudinal approach to assessing MCID achievement, which requires both pre- and posttreatment scores.^1^

The PASS is best calculated by correlating the outcome with an anchor question assessing symptom acceptability.^48^ Patients are commonly asked the following binomial (yes/no) anchor question: “Taking into account all the activities you have during your daily life, your level of pain, and also your functional impairment, do you consider that your current state is satisfactory?”^88^ The PASS threshold can then be determined through different calculation methods, with the most frequent method being the receiver operating characteristic (ROC) curve analysis, which identifies the threshold based on the optimal level of both sensitivity and specificity on the ROC curve.^1,88^

Although the MCID and PASS reflect different concepts, these metrics are complementary and represent tangible and clinically relevant treatment targets. At the individual level, both metrics are clinically relevant for patients and can be used to express results of clinical trials as the proportion of patients who consider themselves better (ie, MCID achievement) or well (ie, PASS achievement) in the different treatment arms.^10,11,88^ The MCID can also provide critical information to researchers in the design of studies in terms of adequate sample size and power calculations.^10,11,81^

Given the importance of the MCID and PASS for the interpretation of PROMs and the increasing use of these metrics in the arthroscopic knee surgery literature, a comprehensive review of all available MCID and PASS thresholds for all available PROMs is warranted.^76^ As such, the purpose of this systematic review is to provide a summary of available literature on MCID and PASS thresholds for PROMs across common primary arthroscopic knee sports surgeries. In particular, this review compiles the calculated MCID and PASS values across all PROMs along with the calculation methods used.

## Methods

This systematic review was conducted based on the Preferred Reporting Items for Systematic Reviews and Meta-Analyses guidelines.^60^

### Search Strategy

The MEDLINE, Embase, and Cochrane databases were queried from inception through January 13, 2025, to identify studies calculating MCID or PASS values of PROMs after common primary arthroscopic sports surgeries of the shoulder, hip, or knee joints and then refined specifically for patients undergoing arthroscopic knee surgery. The full search criteria can be found in Appendix 1. Relevant knee procedures included arthroscopic surgeries of the ACL (eg, ACL reconstruction, ACL repair), meniscus (eg, meniscal repair, partial meniscectomy), or cartilage (eg, knee cartilage repair, microfracture), given that they are among the most commonly performed arthroscopic knee surgeries.^7,29,41,90^

Inclusion criteria were (1) calculation of new MCID or PASS value of a PROM, (2) relevant common primary sports medicine procedure as defined a priori (ie, ACL procedures, meniscus procedures, and cartilage procedures), (3) arthroscopic knee surgery, and (4) full report of outcomes and use of statistical methods. Exclusion criteria included (1) studies not available in English, (2) revision procedures, (3) nonoperative interventions, (4) open procedures, and (5) case reports, reviews, meta-analyses, nonhuman studies, biomechanical studies, technique articles, unpublished studies, and conference proceedings.

### Study Screening

Two authors (D.K. and G.S.) independently screened the titles, abstracts, and full texts using the above inclusion and exclusion criteria. Any discrepancies in inclusion/exclusion were carried to the next round of screening to ensure thoroughness. Remaining conflicts were resolved by consensus opinion. References of all included studies and relevant reviews were further screened to capture any additional studies that may have been missed in the initial search.

### Assessment of Study Quality

Risk of bias and quality of all nonrandomized studies were assessed using the Risk of Bias In Non-randomized Studies–of Interventions (ROBINS-I) tool.^77^ The ROBINS-I tool evaluates the risk of bias across 7 domains. The response options for each domain include (1) low risk of bias, (2) moderate risk of bias, (3) serious risk of bias, (4) critical risk of bias, and (5) no information on which to base a judgment. The overall risk of bias is judged as low if all domains are judged to be at low risk of bias, moderate if all domains are judged to be at low or moderate risk of bias, serious if at least 1 domain is judged to be at serious risk of bias, critical if at least 1 domain is judged to be at critical risk of bias, or no information on which to base a judgment if there is a lack of information in 1 or more key domains and no clear indication that the study is at serious or critical risk of bias.

The risk of bias and quality of all randomized controlled trials were assessed using the Cochrane Risk of Bias 2 (RoB 2) tool.^78^ The Cochrane RoB 2 tool is structured into 5 domains. The possible risk of bias judgments for each domain includes (1) low risk of bias, (2) some concerns, and (3) high risk of bias. The overall risk of bias is judged to be low if all domains are judged to be at low risk of bias, some concerns if at least 1 domain is judged to raise some concerns, or high if at least 1 domain is judged to be at high risk of bias or if multiple domains have some concerns in a way that substantially lowers confidence in the result.

### Data Extraction

Data from each study were extracted into predetermined tables using Google Sheets by 2 reviewers (D.K. and G.S.) and further reviewed by a third author (D.L.L.). Extracted data included author, year of publication, number of participants, type of diagnosis, surgical treatment, length of follow-up, PROMs for which MCID or PASS values were calculated, statistical method (ie, anchor-based or distribution-based method), and calculation method. For MCID values calculated using anchor-based methods, anchor information was also extracted, including the anchor domain (eg, global rating of change), whether the anchor question was reported, the anchor scale, the specific groups compared on the anchor, the number of participants included in threshold calculations, and the correlation between change in the PROM and the anchor.

### Outcomes

The primary outcomes of this study were MCID and PASS thresholds for PROMs after arthroscopic knee surgery. Calculated MCID and PASS values were aggregated by surgical treatment, PROM instrument, and statistical method.

### Statistical Analysis

MCID and PASS thresholds were described using a range, and categorical study characteristics were summarized using frequencies.

## Results

### Study Characteristics and Quality

**Figure 1.**
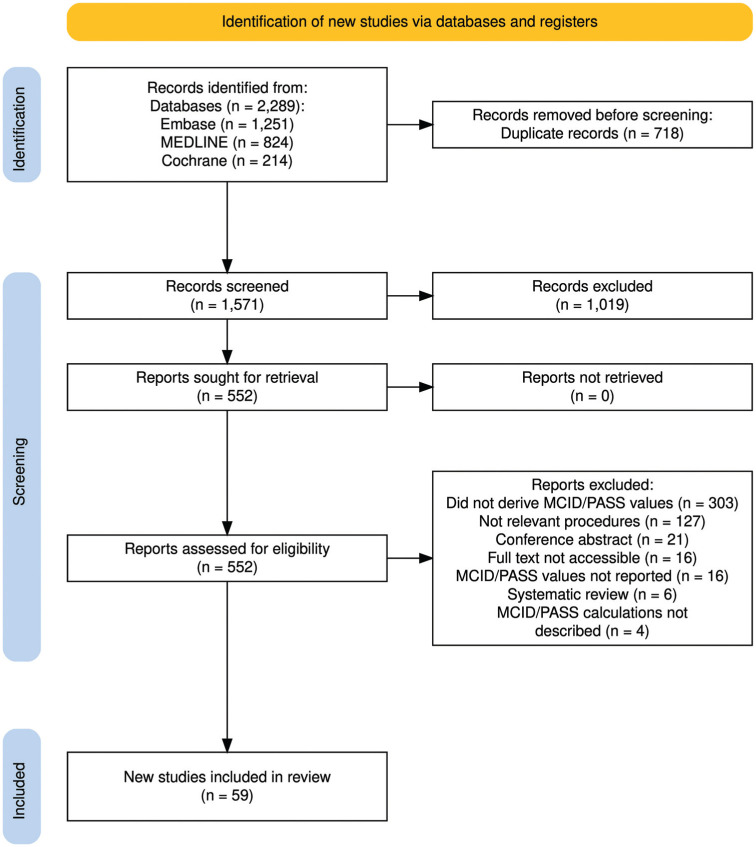
The Preferred Reporting Items for Systematic Reviews and Meta-Analyses diagram of the literature search and study selection.

Of the 1571 studies that were identified, 59 met inclusion criteria and were therefore included in this review (Figure 1).* The results of the risk of bias and quality assessment of the 56 nonrandomized studies included using the ROBINS-I tool are displayed in Appendix 2. All studies showed a moderate overall risk of bias. The results of the risk of bias and quality assessment of the 3 randomized controlled trials included using the Cochrane RoB 2 tool are presented in Appendix 3. All 3 studies had some concerns overall.

Sample sizes and follow-up periods were highly variable, ranging from 12 to 2318 patients and from 2 weeks to 15 years, respectively. Across all studies, thresholds were calculated for 18 different PROMs, with the International Knee Documentation Committee Subjective Knee Evaluation Form (IKDC) being the most frequently reported measure (Table 1).

**Table 1:** Frequency of Patient-Reported Outcome Measures Used

Outcome Measure	Abbreviation	Number of Studies	Score Range	
			Worse Knee Condition	Better Knee Condition
International Knee Documentation Committee Subjective Knee Evaluation Form	IKDC	34	0	100
Knee injury and Osteoarthritis Outcome Score	KOOS^*a*^	32	0	100
Lysholm Knee Scoring Scale	Lysholm	18	0	100
Patient-Reported Outcomes Measurement Information System	PROMIS^*b*,*c*^	7	20	80
Tegner Activity Scale	Tegner	6	0	10
12-Item Short Form Health Survey	SF-12^*b*^	5	0	100
Visual Analog Scale Pain	VAS Pain	3	100	0
Marx Activity Rating Scale	MARS	3	0	16
Western Ontario and McMaster Universities Osteoarthritis Index	WOMAC	2	0	100
Single Assessment Numeric Evaluation	SANE	2	0	100
Anterior Cruciate Ligament Return to Sport after Injury Scale	ACL-RSI	2	0	100
Forgotten Joint Score-12	FJS-12	2	0	100
Western Ontario Meniscal Evaluation Tool	WOMET	1	0	100
36-Item Short Form Health Survey	SF-36	1	0	100
Pediatric International Knee Documentation Committee Subjective Knee Evaluation Form	Pedi-IKDC	1	0	100
Oxford Knee Score	OKS	1	0	48
Wong-Bakar FACES Pain Rating Scale	FACES Scale	1	10	0
Hospital for Special Surgery Pediatric Functional Activity Brief Scale	HSS Pedi-FABS	1	0	30

a KOOS Physical Function Short Form ranges from 100 (worse knee condition) to 0 (better knee condition).

b PROMIS and SF-12 measures recorded using the T-score metric, with a mean of 50 and a standard deviation of 10 based on the general US population.

c Greater scores indicate better knee condition for PROMIS Physical Function, while lower scores indicate better knee condition for PROMIS Pain Interference and PROMIS Depression.

Thirty-five studies (59%) calculated thresholds for an ACL procedure, 15 studies (25%) for a meniscus procedure, and 8 studies (14%) for a cartilage procedure. ACL procedures included reconstruction and repair; meniscus procedures included allograft transplantation, resection, repair, and partial/total meniscectomy; and cartilage procedures included autologous chondrocyte implantation, repair, microfracture, mosaicplasty, and osteochondral allograft transplantation. Two additional studies (3%) calculated thresholds for a sample that underwent partial medial/lateral meniscectomy, chondroplasty, loose body removal, and/or synovectomy,^39^ as well as a sample that underwent concomitant ACL reconstruction with posterior lateral meniscus root tear repair,^98^ and were grouped separately. The frequency of each procedure is displayed in Table 2.

**Table 2:** Frequency of Procedures Performed^*a*^

Procedure	No. of Studies
ACL-based	35
ACL reconstruction	32
ACL repair	4
Meniscus-based	15
Partial meniscectomy	7
Meniscal allograft transplantation	3
Meniscal repair	2
Meniscal resection and/or repair	2
Meniscectomy	1
Cartilage-based	8
Mosaicplasty	2
Autologous chondrocyte implantation	1
Cartilage repair	1
Microfracture	1
Microfracture or matrix-assisted autologous chondrocyte transplantation	1
Mosaicplasty or osteochondral allograft transplantation	1
Osteochondral allograft transplantation	1
Other	2
Partial medial meniscectomy, partial lateral meniscectomy, chondroplasty, loose body removal, and/or synovectomy	1
ACL reconstruction with posterior lateral meniscus root tear repair	1

a ACL, anterior cruciate ligament.

### MCID Calculation Methods

In total, 52 studies (88%) calculated MCID thresholds. Across these studies, 39 studies (75%) used distribution-based approaches. Calculation methods included half the standard deviation (n = 37 studies, 95%), effect size (n = 2 studies, 5%), one-third the standard deviation (n = 1 study, 3%), standard error (n = 1 study, 3%), and the Spratt method (n = 1 study, 3%). Fifteen studies (29%) used an anchor-based approach to calculate the MCID, with 4 different calculation methods used, including ROC curve analysis (n = 10 studies, 67%), mean change (n = 4 studies, 27%), mean difference (n = 4 studies, 27%), and predictive modeling (n = 2 studies, 13%). Characteristics of studies calculating MCID thresholds for PROMs are displayed in Appendix 4.

Among the 15 studies that used anchor-based approaches to calculate MCID, the most common type of anchor was a global rating of change scale (n = 8 studies, 53%). Single-anchor scales ranged from 5 to 15 points, with 7-point anchors being the most frequently used (n = 6 studies, 40%). Seven studies (47%) reported the correlation between the change in the PROM and the anchor, ranging from 0.32 to 0.79. Anchor details for studies calculating anchor-based MCID values are reported in Appendix 5.

### PASS Calculation Methods

A total of 21 studies (36%) calculated PASS thresholds, with all studies employing anchor-based approaches. Three different calculation methods were used, including ROC curve analysis (n = 18 studies, 86%), predictive modeling (n = 2 studies, 10%), and the 80% specificity method (n = 1 study, 5%). Characteristics of studies calculating PASS thresholds for PROMs can be found in Appendix 6.

### MCID Thresholds for ACL, Meniscus, and Cartilage Procedures

The ranges of MCID values for individual procedures across all reported PROMs are shown in Table 3.

**Table 3:** MCID Threshold Ranges for Individual Procedures Across PROMs^*a*^

Procedure	Instrument	Distribution		Anchor	
		No. of Studies	MCID Range	No. of Studies	MCID Range
ACL reconstruction					
	IKDC	10	7.6-10.5	3	7.1-16.2
	KOOS Total	2	5.3-9.2		
	KOOS Pain	5	7.4-10.6	1	7.9-13.9
	KOOS Symptoms	4	9.3-10.6	1	1.2-5.4
	KOOS Activities of Daily Living	4	8.1-12.6	1	5.1-8.1
	KOOS Sport and Recreation Function	4	13.1-16.8	2	2.5-21.7
	KOOS Knee-Related Quality of Life	3	13.6-14.6	1	21.9-27.3
	Lysholm	8	9.1-15.0		
	PROMIS Physical Function	2	2.6-4.6	1	4.5
	PROMIS Pain Interference	1	−4.0	1	−5.4
	PROMIS Depression	1	−4.9	1	−4.1
	Tegner	4	0.7-1.3	1	0.5
	SF-12 Total	1	5.3		
	SF-12 Physical Component Score	3	4.0-5.2		
	SF-12 Mental Component Score	3	3.1-5.1		
	VAS Pain	2	8.8-11.0		
	MARS	1	1.9		
	SANE	2	14.0		
	Pedi-IKDC	1	9.3		
	OKS	1	5.2		
	HSS Pedi-FABS	1	5.9		
ACL repair					
	IKDC	4	3.8-14.3		
	KOOS Pain	2	5.7-11.6		
	KOOS Symptoms	2	5.6-8.2		
	KOOS Activities of Daily Living	2	4.7-12.2		
	KOOS Sport and Recreation Function	2	5.6-19.0		
	KOOS Knee-Related Quality of Life	1	6.5-7.2		
	Lysholm	3	3.5-8.5		
	Tegner	1	5.2-6.1		
	WOMAC	1	4.6-10.4		
	ACL-RSI	1	12.2		
	FJS-12	1	3.5		
Partial meniscectomy					
	IKDC	1	10.6		
	KOOS Total	1	5.1-7.6		
	KOOS Pain	3	8.8-9.7		
	KOOS Symptoms	3	6.6-9.2		
	KOOS Activities of Daily Living	2	10.2-11.0		
	KOOS Sport and Recreation Function	3	11.8-14.9		
	KOOS Knee-Related Quality of Life	3	9.0-15.6		
	KOOS Physical Function Short Form	1	−8.2		
	KOOS Joint Replacement	1	10.7		
	PROMIS Physical Function	2	2.5-3.8	1	2.1
	PROMIS Pain Interference	1	−3.3		
	PROMIS Depression	1	−5.3		
	WOMAC Pain	1	9.1-9.6		
	WOMAC Stiffness	1	12.1-12.5		
	WOMAC Physical Function	1	8.6-9.3		
	SF-36 Pain	1	13.0-13.5		
	SF-36 Physical Functioning	1	14.4-14.7		
	SF-36 General Health	1	5.7-6.5		
Meniscal allograft transplantation					
	IKDC	3	9.9-12.1		
	KOOS Pain	2	9.9-11.0		
	KOOS Symptoms	3	9.7-11.0		
	KOOS Activities of Daily Living	2	9.5-10.5		
	KOOS Sport and Recreation Function	2	13.3-16.2		
	KOOS Knee-Related Quality of Life	2	13.6-14.6		
	Lysholm	2	10.3-12.3		
Meniscal repair					
	IKDC	1	10.9		
	KOOS Pain	1	11.8		
	KOOS Symptoms	1	12.3		
	KOOS Activities of Daily Living	1	11.4		
	KOOS Sport and Recreation Function	1	16.7		
	KOOS Knee-Related Quality of Life	1	16.9		
	FACES Scale	1	−1.5		
Meniscectomy					
	IKDC			1	10.4
	KOOS Pain			1	9.7
	KOOS Symptoms			1	10.4
	KOOS Activities of Daily Living			1	10.5
	KOOS Sport and Recreation Function			1	14.7
	KOOS Knee-Related Quality of Life			1	13.2
	KOOS Physical Function Short Form			1	−8.5
	KOOS Joint Replacement			1	10.9
Autologous chondrocyte implantation					
	IKDC			1	12.6-16.4
	KOOS Pain			1	11.1-18.8
	KOOS Symptoms	1	3.6-8.4		
	KOOS Activities of Daily Living			1	10.3-17.3
	KOOS Sport and Recreation Function			1	15.0-18.6
	KOOS Knee-Related Quality of Life			1	12.8-19.6
	Lysholm	1	4.2-10.5		
	SF-12 Physical Component Score			1	6.2-8.2
	SF-12 Mental Component Score	1	1.9-4.6		
Microfracture					
	IKDC	1	10.1-16.7		
	KOOS Pain	1	8.9-10.5		
	KOOS Symptoms	1	8.3-10.9		
	KOOS Activities of Daily Living	1	8.7-11.2		
	KOOS Sport and Recreation Function	1	14.9-15.2		
	KOOS Knee-Related Quality of Life	1	12.9-14.3		
Mosaicplasty					
	IKDC	1	9.3		
	KOOS Symptoms	1	7.4		
	Lysholm	1	6.6-8.4		
	VAS Pain	1	12.9		
	MARS	1	2.5		
Osteochondral allograft transplantation					
	IKDC			1	9.8
	KOOS Pain			1	16.7
	KOOS Symptoms	1	2.5-6.3		
	KOOS Activities of Daily Living	1	3.7-9.2		
	KOOS Sport and Recreation Function			1	25.0
	KOOS Knee-Related Quality of Life	1	3.7-9.3		
	Lysholm	1	3.7-9.2		
	SF-12 Physical Component Score	1	1.7-4.2		
	SF-12 Mental Component Score	1	1.8-4.6		

a MCID threshold ranges only included for single procedures. ACL, anterior cruciate ligament; ACL-RSI, Anterior Cruciate Ligament Return to Sport after Injury Scale; FACES, Wong-Baker FACES Pain Rating Scale; HSS Pedi-FABS, Hospital for Special Surgery Pediatric Functional Activity Brief Scale; IKDC, International Knee Documentation Committee Subjective Knee Evaluation Form; KOOS, Knee injury and Osteoarthritis Outcome Score; MARS, Marx Activity Rating Scale; MCID, minimal clinically important difference; OKS, Oxford Knee Score; Pedi-IKDC, Pediatric International Knee Documentation Committee Subjective Knee Evaluation Form; PROM, patient-reported outcome measure; PROMIS, Patient-Reported Outcomes Measurement Information System; SANE, Single Assessment Numeric Evaluation; SF-12, 12-Item Short Form Health Survey; SF-36, 36-Item Short Form Health Survey; VAS, visual analog scale; WOMAC, Western Ontario and McMaster Universities Osteoarthritis Index.

In total, 29 studies calculated MCID thresholds for PROMs after ACL procedures (Appendix 7), with IKDC (n = 16 studies) being the most commonly reported instrument. MCID thresholds for IKDC following ACL reconstruction ranged from 7.6 to 10.5 using distribution-based methods and 7.1 to 16.2 using anchor-based methods. MCID thresholds after ACL repair ranged from 3.8 to 14.3 using distribution-based methods.

Fourteen studies calculated MCID thresholds for PROMs after meniscus procedures (Appendix 8). The Knee injury and Osteoarthritis Outcome Score (KOOS) symptoms subscale (n = 10 studies) was the most commonly reported measure. MCID thresholds calculated using distribution-based methods ranged from 9.7 to 11.0 after meniscal allograft transplantation, 12.3 after meniscal repair, and 6.6 to 9.2 after partial meniscectomy. Anchor-based MCID thresholds ranged from 6.4 to 8.0 after meniscal resection and/or repair and 10.4 after meniscectomy.

Eight studies calculated MCID thresholds for PROMs after cartilage procedures (Appendix 9). IKDC (n = 6 studies) was one of the most frequently reported instruments. Distribution-based MCID thresholds ranged from 10.1 to 16.7 after microfracture and 9.3 after mosaicplasty. Anchor-based MCID thresholds ranged from 12.6 to 16.4 after autologous chondrocyte implantation, 9.2 after cartilage repair, 17.0 after mosaicplasty or osteochondral allograft transplantation, and 9.8 after osteochondral allograft transplantation.

Calculated MCID thresholds, aggregated by procedure, PROM, and statistical method, following other procedures are displayed in Appendix 10.

### PASS Thresholds for ACL, Meniscus, and Cartilage Procedures

The ranges of PASS values for individual procedures across all reported PROMs are displayed in Table 4.

**Table 4:** PASS Threshold Ranges for Individual Procedures Across PROMs^*a*^

Procedure	Instrument	No. of Studies	PASS Range
ACL reconstruction			
	IKDC	5	66.7-80.5
	KOOS Pain	4	80.6-88.9
	KOOS Symptoms	4	45.7-78.6
	KOOS Activities of Daily Living	4	77.2-100.0
	KOOS Sport and Recreation Function	4	70.0-80.0
	KOOS Knee-Related Quality of Life	4	50.0-75.0
	KOOS Physical Function Short Form	1	18.6
	KOOS Joint Replacement	1	76.3
	Lysholm	1	86.0
	PROMIS Physical Function	1	56.0
	PROMIS Pain Interference	1	49.7
	ACL-RSI	1	40.0
	Pedi-IKDC	1	66.2
ACL repair			
	IKDC	2	73.6-88.6
	KOOS Pain	2	83.3-91.7
	KOOS Symptoms	2	75.0-85.7
	KOOS Activities of Daily Living	2	83.8-99.0
	KOOS Sport and Recreation Function	2	75.0-89.0
	KOOS Knee-Related Quality of Life	2	62.5-81.3
	Lysholm	2	89.0-90.0
	Tegner	1	8.3-8.9
	WOMAC	1	80.3-93.2
	ACL-RSI	1	54.2
	FJS-12	1	68.8
Partial meniscectomy			
	IKDC	2	56.2-57.9
	KOOS Pain	2	76.4-81.6
	KOOS Symptoms	2	64.3-71.4
	KOOS Activities of Daily Living	2	82.4-89.0
	KOOS Sport and Recreation Function	2	55.6-71.0
	KOOS Knee-Related Quality of Life	2	46.9-51.0
	KOOS Physical Function Short Form	1	26.2
	KOOS Joint Replacement	1	68.3
	PROMIS Physical Function	1	46.1
	MARS	1	7.0
	WOMET	1	58.5
Meniscal allograft transplantation			
	IKDC	2	36.0-55.6
	KOOS Pain	1	70.7
	KOOS Symptoms	2	60.8-73.0
	KOOS Activities of Daily Living	1	90.3
	KOOS Sport and Recreation Function	1	47.4
	KOOS Knee-Related Quality of Life	2	40.5-53.0
	Lysholm	2	66.5-74.5
Meniscal repair			
	IKDC	1	69.0
	KOOS Pain	1	80.6
	KOOS Symptoms	1	75.0
	KOOS Activities of Daily Living	1	92.7
	KOOS Sport and Recreation Function	1	80.0
	KOOS Knee-Related Quality of Life	1	56.3
Meniscectomy			
	IKDC	1	57.9
	KOOS Pain	1	76.4
	KOOS Symptoms	1	71.4
	KOOS Activities of Daily Living	1	89.0
	KOOS Sport and Recreation Function	1	55.6
	KOOS Knee-Related Quality of Life	1	46.9
	KOOS Physical Function Short Form	1	26.2
	KOOS Joint Replacement	1	68.3
Microfracture			
	IKDC	1	51.2-68.7
	KOOS Pain	1	70.8-80.6
	KOOS Symptoms	1	76.8-82.1
	KOOS Activities of Daily Living	1	88.2-94.1
	KOOS Sport and Recreation Function	1	37.5-62.5
	KOOS Knee-Related Quality of Life	1	46.9-53.1

a PASS threshold ranges only included for single procedures. ACL, anterior cruciate ligament; ACL-RSI, Anterior Cruciate Ligament Return to Sport after Injury Scale; FJS-12, Forgotten Joint Score-12; IKDC, International Knee Documentation Committee Subjective Knee Evaluation Form; KOOS, Knee injury and Osteoarthritis Outcome Score; MARS, Marx Activity Rating Scale; PASS, patient acceptable symptom state; Pedi-IKDC, Pediatric International Knee Documentation Committee Subjective Knee Evaluation Form; PROM, patient-reported outcome measure; PROMIS, Patient-Reported Outcomes Measurement Information System; WOMAC, Western Ontario and McMaster Universities Osteoarthritis Index; WOMET, Western Ontario Meniscal Evaluation Tool.

Ten studies calculated PASS thresholds for PROMs after ACL procedures (Appendix 11), with IKDC (n = 7 studies) being the most commonly reported instrument. PASS thresholds for IKDC ranged from 66.7 to 80.5 after ACL reconstruction and 73.6 to 88.6 after ACL repair.

Nine studies calculated PASS thresholds for PROMs after meniscus procedures (Appendix 12). The KOOS knee-related quality of life subscale (n = 8 studies) was one of the most reported instruments. PASS thresholds ranged from 40.5 to 53.0 after meniscal allograft transplantation, 51.3 to 53.6 after meniscal resection and/or repair, 46.9 to 51.0 after partial meniscectomy, 56.3 after meniscal repair, and 46.9 after meniscectomy.

Two studies calculated PASS thresholds for PROMs after cartilage procedures (Appendix 13), with IKDC (n = 2 studies) being one of the most frequently reported measures. PASS thresholds ranged from 51.2 to 68.7 after microfracture and 62.1 after cartilage repair.

## Discussion

This study systematically investigated the MCID and PASS values of all available PROMs for arthroscopic surgeries addressing ACL tears, meniscus injuries, and cartilage damage. This review is informative as it can help guide investigators in selecting MCID and PASS values to use as clinically relevant treatment targets for patients undergoing arthroscopic knee surgeries. One of the limitations of PROMs is that they are often expressed as continuous data at the group level, which may be difficult to interpret and translate to the responses of individual patients.^82,89^ Instead of just comparing treatment arms using means and standard deviations at the group level, this study helps investigators assess patients in clinical trials at the individual level by comparing the proportion of patients achieving MCID and PASS thresholds after each treatment.^10,11,88^ The review of MCID thresholds can also inform the design of future studies requiring sample size and power calculations.^10,11,81^

One of the main findings of the current review is that of the 52 studies that calculated MCID thresholds, only 15 used anchor-based approaches, while 39 used distribution-based approaches. The discrepancy in studies that used anchor-based versus distribution-based approaches is significant, given that anchor-based approaches are preferred as the primary method for calculating the MCID.^16,19,62,74^ Distribution-based approaches should be considered as supportive or secondary measures because they do not directly reflect the patient's perception of improvement, while anchor-based approaches allow patients to directly quantify the importance of change.^16,19,55,62,74^ Future studies should prioritize using anchor-based MCID calculations despite the increased investigator and patient burden relative to distribution-based approaches.

Among the 15 studies that implemented anchor-based approaches to calculate MCID values, 4 different calculation methods were used, including ROC curve analysis, mean change, mean difference, and predictive modeling. The most common method was ROC curve analysis, which identifies thresholds based on the optimal level of both sensitivity and specificity.^23,85^ Among the anchor-based methods, ROC curve analysis is considered optimal for calculating MCID values to be applied at the individual level because it aims to minimize misclassification of patients.^23,85^ The mean change method, in particular, has been described as not being useful for individual-level applications because of the use of group means to establish the MCID.^23,85^ An anchor-based approach using ROC curve analysis is therefore the recommended primary method for MCID quantification.

Anchor details for MCID values calculated using anchor-based methods were also reviewed in this study. The most common type of anchor was a global rating of change scale; however, previous work reported that domain-specific anchors had higher construct validity for determining the MCID for PROMs focused on a specific domain than global anchors.^94^ Investigators should therefore consider using domain-specific anchors for PROMs that assess specific domains. For example, when calculating MCID values for KOOS subscales, the utilization of a separate subscale-specific anchor for each subscale should be prioritized over the use of a global anchor that assesses overall change. It was also found that the unchanged and minimally improved groups based on the anchor were defined differently between studies. Some studies included patients who reported being “worse” in the unchanged group and those who reported being “a good deal better” or “fully recovered” in the minimally improved group, which may both result in higher MCID thresholds.^79^ Despite recommendations that anchor information, including the type of anchor, the anchor question, the anchor scale, the specific groups compared on the anchor, the number of participants included in MCID calculations, and the correlation between the change in the PROM and the anchor, should be reported,^9^ only 6 of 15 studies provided all details. The correlation between change in the PROM and the anchor is particularly important to consider, as previous studies have suggested using the correlation as a criterion to assess the credibility of MCID values, with values considered credible if the correlation is ≥0.4 and values considered questionable if the correlation is <0.4 or if no correlation is reported.^18,37,79^ Investigators calculating anchor-based MCID values should select interpretable anchors that correlate at least moderately with the PROM, with stronger associations contributing to more credible threshold estimates, and report all relevant anchor information.

When comparing the reported MCID values, considerable variability was observed. For example, MCID values ranged from 7.1 to 16.2 and 3.8 to 14.3 for the IKDC instrument for ACL reconstruction and ACL repair, respectively. The reported MCID values likely depend on the calculation method used, surgical treatment, study population, underlying pathology, length of follow-up, and baseline pain and function.^1,14,15,49,84^ The heterogeneity in MCID values presents a challenge to investigators when selecting the appropriate value to use for power calculations or when calculating the proportion of patients achieving the MCID across treatment arms in clinical trials. MCID values should be viewed as context specific.^49,79^ As such, investigators must review the characteristics of the study sample in which the values were calculated (eg, age, baseline scores, diagnosis, intervention, length of follow-up) to determine whether they match the context of its intended application. Investigators should therefore prioritize selecting MCID values derived using appropriate methods and calculated for their own study population or in contexts that most closely resemble it.

In total, fewer studies reported PASS values (n = 21 studies) than MCID values (n = 52 studies) for PROMs after arthroscopic knee surgery. The relatively lower reporting of PASS requires consideration, given that previous work has described that it is more important to patients if they feel good (ie, achieve PASS) rather than if they feel better (ie, achieve MCID).^89^ In addition, an advantage of utilizing PASS is the cross-sectional approach used to assess PASS achievement, which allows investigators to determine whether patients are satisfied with their current state at any point in time without requiring pretreatment scores. Future studies should consider reporting PASS alongside MCID as they are different, but complementary, metrics.

Most studies calculating PASS thresholds in this review used ROC curve analysis. Two of the most commonly used statistical approaches in the literature for deriving the PASS include ROC curve analysis and the 75th percentile approach (ie, 75th percentile of the cumulative distribution of the outcome for patients who rated their state as satisfactory).^1,48,53^ The ROC approach may provide PASS estimates somewhat lower than the estimates identified using the 75th percentile approach^53^; however, no studies implemented the 75th percentile approach in this review, and therefore, these methods could not be compared. The ROC approach may minimize misclassification of patients as satisfied or unsatisfied, although further research is needed to compare different calculation methods and determine the most optimal approach for calculating PASS thresholds.

Like the variability in reported MCID values, heterogeneity was also observed in PASS values. For example, PASS values for the IKDC instrument ranged from 66.7 to 80.5 after ACL reconstruction and 73.6 to 88.6 after ACL repair. Although PASS values are likely influenced by the same factors as MCID values, studies have shown that PASS values may be more stable over time and less strongly influenced by age, disease duration, and sex.^1,46,87^ When selecting which PASS values to use as clinically relevant benchmarks in studies, investigators should apply PASS cutoffs that were calculated in similar contexts.

### Limitations

Several limitations of this review should be considered. First, the wide heterogeneity in research methods across studies (eg, participant characteristics, follow-up periods, calculation methods) created difficulties in identifying clear patterns to explain the variation in reported MCID and PASS values. Second, MCID and PASS values were both described using a range, which can be imprecise. Finally, relatively few studies calculated MCID and PASS values for some PROMs and procedures, and therefore, additional research is needed to refine these thresholds.

## Conclusion

This study systematically reviewed and reported the MCID and PASS values of all available PROMs for ACL, meniscus, and cartilage surgery. The heterogeneity observed in reported MCID and PASS values suggests that these metrics should be viewed as context specific. While most studies in this review used distribution-based calculations to derive MCID values, anchor-based calculations should be prioritized in future studies. Overall, the information in this study will allow investigators to use appropriate clinically relevant thresholds for designing randomized controlled trials, comparing the proportion of patients achieving a meaningful improvement and satisfactory state across different treatment arms to evaluate effectiveness, and establishing patient expectations for recovery.

## Footnotes

* References 2-6, 10, 12, 13, 17, 20-22, 24, 26-28, 30-35, 38-40, 42-45, 47, 50-52, 54, 56, 58, 59, 61, 63-73, 75, 80, 83, 86, 91-93, 96-98.
